# Qualifying Osteogenic Potency Assay Metrics for Human Multipotent Stromal Cells: TGF-β2 a Telling Eligible Biomarker

**DOI:** 10.3390/cells9122559

**Published:** 2020-11-29

**Authors:** Augustin M. Ofiteru, Diana F. Becheru, Sami Gharbia, Cornel Balta, Hildegard Herman, Bianca Mladin, Mariana Ionita, Anca Hermenean, Jorge S. Burns

**Affiliations:** 1 Faculty of Medical Engineering, University Politehnica of Bucharest, Gh Polizu 1-7, 011061 Bucharest, Romania; diana.becheru@yahoo.com (D.F.B.); mariana.ionita@polimi.it (M.I.); 2 Faculty of Applied Chemistry and Materials Science, University Politehnica of Bucharest, Gh Polizu 1-7, 011061 Bucharest, Romania; 3 “Aurel Ardelean” Institute of Life Sciences, Vasile Goldis Western University of Arad, 86 Rebreanu, 310414 Arad, Romania; samithgh2@hotmail.com (S.G.); baltacornel@gmail.com (C.B.); hildegard.i.herman@gmail.com (H.H.); biancaonitamaria@gmail.com (B.M.); anca.hermenean@gmail.com (A.H.); 4 Department of Life Sciences and Biotechnology, University of Ferrara, 44121 Ferrara, Italy

**Keywords:** bone, multipotent stromal cells, stem cells, adipose tissue, bone marrow, gene expression, *TGFB2*, TGF-β2, decorin, osteogensys, potency assay

## Abstract

Potency assays are critical for regenerative medicine, addressing the known challenge of functional heterogeneity among human multipotent stromal cells (hMSC). Necessary laboratory cell expansion allows analysis before implantation in the patient. Levels of induction of five signature gene biomarkers, *ALPL*, *COL1A2*, *DCN*, *ELN* and *RUNX2*, constituted a previously reported proof-of-principle osteogenic potency assay. We tested assay modification to enhance reproducibility using six consistent bone marrow derived hBM-MSC and explored applicability to three adipose tissue derived hAT-MSC. Using a potent proprietary osteogenic induction factor, the GUSB/YWAHZ reference gene pair provided real time PCR consistency. The novel assay conditions supported the concept that genes encoding extracellular matrix proteins one week after osteogenic induction were informative. Nonetheless, relatively low induction of *COL1A2* and *ELN* encouraged search for additional biomarkers. *TGFB2* mRNA induction, important for osteogenic commitment, was readily quantifiable in both hBM-MSC and hAT-MSC. Combined with *DCN*, *TGFB2* mRNA induction data provided discriminatory power for resolving donor-specific heterogeneity. Histomorphometric decorin and TGF-β2 protein expression patterns in eight-week heterotopic bone implants also discriminated the two non-bone-forming hMSC. We highlight progress towards prompt osteogenic potency assays, needed by current clinical trials to accelerate improved intervention with enhanced stem cell therapy for serious bone fractures.

## 1. Introduction

Seeking to ameliorate an enormous healthcare burden from bone morbidities through tissue engineering [1], use of autologous culture-expanded osteoprogenitor cells grown on porous bioceramic scaffolds could substantially improve the repair of large defects in long bones [2]. This approach has been supported by positive outcomes from early stage clinical trials [3] including use of bone marrow derived human Multipotent Stromal Cells (hMSC) as advanced therapy medicinal products (ATMP) for non-union bone fractures [4]. Nonetheless, donor-related hMSC heterogeneity [5] can hinder therapeutic efficacy [6] and a consensus view recommends rigorous and thorough supportive protocols [7]. This is particularly relevant for a cell-based osteogenic potency assay to confirm appropriate biological activity of the specific therapeutic cells, a required release criterion before final marketing authorization of the patient treatment. International Conference on Harmonization guidelines (ICH 6QB) endorsed by the European Medical Agency (EMA) stipulated a quantitative measure of biological activity linked to the relevant biological properties based on the intended biological effect, ideally related to the clinical response [8].

An implemented timeline of 21 days for Good Manufacturing Practice (cGMP) cell expansion in the two-step protocol for osteogenic cell therapy [9] highlighted need for a prompt potency assay method to test biological function. Human MSC can be differentiated in vitro by supplementing a growth maintenance medium with osteogenic induction factors [10] that stimulate a chronological series of long-appreciated stepwise cellular changes: a reduced proliferation, extracellular matrix (ECM) maturation and subsequent mineralization [10,11,12]. Although extensively researched, the complex series of molecular mechanisms governing these differentiation pathway processes remain to be fully understood. Nonetheless, live monitoring of hMSC differentiation with mRNA-based probes revealed changes in the ratio of master transcription factors Runx2/Sox9 correlated with induction of osteogenic differentiation [13]. Stepwise changes in ECM are fundamental for bone formation [14] and ECM from early stages of osteogenic differentiation promoted in vitro osteogenic differentiation [15]. Cellular models comparing telomerized hBM-MSC subclones of contrasting heterotopic bone-forming capacity indicated that ECM biomarkers were important for prospective identification of bone formation [16].

Among several potency assay approaches [17], a survey of candidate early inducible biomarkers identified a set of five indicative “signature genes”—differentiation-induced expression of the transcription factor runt related transcription factor 2 (RUNX2) and of four genes encoding ECM proteins; tissue-nonspecific alkaline phosphatase (ALPL); collagen type I alpha 2 chain (COL1A2); decorin (DCN); and elastin (ELN)—that together provided a one-week osteogenic induction assay that clustered donor-specific primary cGMP cultures of hBM-MSC according to their bone-forming potential [18].

Progress in the material sciences favoring development of dedicated biosensors [19] has raised emphasis on standardization for robust and scalable potency assay conditions. Consequently, we qualified the above-mentioned signature gene potency assay, performing a cross-laboratory comparison using the same previously described bone marrow derived cGMP-hBM-MSC populations (Donors #1–#6) [16]. Given interest in enhancing bone formation using more readily sourced multipotent stromal cells derived from adipose tissue (AT) [20,21,22,23,24,25], we extended the study to also include three hAT-MSC populations (Donors #7–#9) grown under the same cGMP culture conditions.

Potency assay standardization may be improved through careful choice of suitable polymerase chain reaction (PCR) normalization reference genes [26] and use of effective scalable reagents for osteogenic induction. Thus, we tested reference genes for glucuronidase beta (*GUSB*) and the gene encoding 14-3-3 protein zeta/delta (*YWAHZ*), reported to be appropriate for hMSC differentiation-related gene expression [27] in comparison to the previously used actin beta (*ACTB*) [18]. A proprietary medium, specifically formulated for prompt hMSC differentiation [28,29], was a suitable induction reagent for a rapid osteogenic potency assay. Using the updated potency assay conditions of a qualified reference gene pair and potent osteogenic induction medium, we found the signature gene biomarkers segregated by having high (*ALPL* and *DCN*) or low (*COL1A2*, *ELN*, *RUNX2*) levels of induction. Since biomarkers induced by only low levels may prove problematic for biosensor development, we sought an additional biomarker.

A bioinformatic protein interaction network between the potency assay signature gene products and the transforming growth factor beta1 (TGF-β1) pathway [18] was consistent with the latter’s importance in fracture healing [30] and osteoblastic differentiation [31]. Recent studies of osteogenic or adipogenic differentiation have also implicated the transforming growth factor beta family member TGF-β 2 in hMSC osteogenic lineage commitment [32]. Thus, we examined the induction profile concerning the relatively less-studied TGF-β2 isoform as a novel signature gene candidate.

*TGFB2* gene induction levels were similar to robust profiles for *ALPL* and *DCN*. Providing new qualities to the signature gene repertoire, the timing of *TGFB2* induction was uniquely capable of distinguishing hBM-MSC from hAT-MSC. Our cross-laboratory osteogenic potency assay comparison highlighted the complex nature of hMSC, the usefulness of ECM biomarkers in early phases of osteogenic differentiation and introduced a novel signature gene candidate as a biomarker with broad implications for biosensor and ATMP development.

## 2. Materials and Methods

### 2.1. Cell Culture

Bone marrow derived human Multipotent Stromal Cell (hBM-MSC) populations, independently derived from six donors, designated #1 to #6, and adipose tissue derived human Multipotent Stromal Cell (hAT-MSC) populations derived from three donors, designated #7 to #9, were kindly provided by the University Hospital of Modena and Reggio Emilia (UNIMORE). Cell isolation using previously described methods for hBM-MSC from the iliac crest [33] or hAT-MSC from lipoaspirates [34] followed written informed consent from the donors within the EU FP7-Health REBORNE project, with ethics approval as published [4]. Our experimental procedures were first approved by the Ethics and Academic Integrity Committee of University Politehnica of Bucharest (Approval no. 22083/07.11.2017).

Thawed cryopreserved cells were seeded at 6 × 10^3^ cells/cm^2^ in T75 flasks (U-Shaped Canted Neck Cell Culture Flask with Vent Cap, Corning, NY, USA), with maintenance medium (MM), Alpha Modified Minimum Essential Medium Eagle (αMEM), (M4526, Sigma Aldrich, St. Louis, MO, USA), supplemented with 5% platelet lysate (PLTMax), (Human Platelet Lysate, SCM141, Sigma Aldrich), 1% Glutamax (Glutamax I, 200 mM, Thermo Fisher Scientific, Boston, MA, USA), 2 IU/mL Heparin Sodium Salt (H3149, Sigma Aldrich) and 10 μg/mL Ciprofloxacin (PHR1044, Sigma Aldrich). Cultures were maintained in humidified cell culture incubators (Steri-cycle i160, Thermo Fisher Scientific, Boston, MA, USA) at 37 °C, 5% CO_2_ with medium replenishment every 2–3 days. At 80% confluence, the cells were detached with TrypLE Express 1X (12563011, Gibco™, Invitrogen, Belgium) and counted using an automated cell counter, Scepter 2.0 (Merck, NJ, USA), with 60 μM sensors.

The calculus for the population doubling number (*PD*) was based on the formula:

$$PD=\log_{10}\left(C/H\right)/\log_{10}2,$$

where (*C*) is number of cells seeded and (*H*) is the number of cells harvested.

Cumulative population doubling (*CPD*) was calculated using the formula:

$$CPD=\sum_{i=1}^{n}\mathrm{PD}i,$$

where (*n*) is the last passage number.

### 2.2. Induction of Ex Vivo Osteogenic Differentiation

Cells for total RNA extraction were seeded at a density of 3 × 10^4^/cm^2^ in T25 cell culture flasks (Rectangular Canted Neck Cell Culture Flask with Vent Cap, Corning, NY, USA), using standard MM. Upon reaching 80–90% confluence (usually 2–3 days), the medium was replaced with proprietary osteogenic medium (OM), (OsteoMAX-XF™, SCM121, Sigma Aldrich), supplemented with 4% PLTMax. The cultures were maintained for one and two weeks, renewing the medium every 2–3 days and extracted total RNA was used to generate the corresponding cDNA library.

For monitoring matrix mineralization, hMSC were seeded in 24-well flat bottom culture plates (Gibco™, Invitrogen, Belgium), at a density of 3 × 10^4^ cells/well, maintained in standard culture conditions for 24 h in MM, than the medium was replaced with OM supplemented with 4% PLTMax. Parallel control samples were cultured in MM. Confluent monolayers cultures in plates were fixed and stained with Alizarin Red S and Von Kossa at 7, 14 and 21 days post osteogenic induction, in order to assess the extent of matrix mineralization.

### 2.3. Matrix Mineralization Assays

Two methods, namely, Alizarin Red S (ALZ) for direct staining of calcium deposits [35] and Von Kossa (VK) for indirect staining of calcium phosphate [36], were used to determine the extent of ECM mineralization during osteoblastic differentiation. The biomineralization process was monitored at 7, 14 and 21 days in OM culture conditions. For ALZ stain, cells were washed with phosphate buffer saline (PBS), (PBS 1X, -Ca^2+^/-Mg^2+^, Gibco™, Invitrogen, Belgium), fixed with ice-cold 10% formalin (F-5554-4L, Sigma Aldrich) for 10 min at room temperature (RT), washed with ultrapure distilled water (Gibco™, Invitrogen, Belgium) and stained with 1.5%, pH 4.2 aqueous solution of Alizarin Red S (A5533, Sigma Aldrich) for 10 min. Stained wells were rinsed twice with ultrapure distilled water and completely dried in an air flow-hood, before sample visualization with an inverted phase contrast microscope (DMIL LED Fluo model with DFC450-C capture system, Leica, Germany). For quantification of total mineralization of the cell monolayer, the ALZ dye was eluted by adding 500 μL/well of hexadecylpyridinium chloride monohydrate 10% *w*/*w* (C9002, Sigma Aldrich). After 15 min, the eluted dye was quantified using an UV-Vis spectrophotometer, DeNovix DS-11 (DeNovix, Wilmington, DE, USA), at 562 nm.

VK staining was performed at the same experimental time points as ALZ. Cells were washed with PBS, fixed with 10% formalin for 10 min and rinsed with ultrapure distilled water. The plates were incubated for 30 min with 2.5% aqueous solution of silver nitrate (85193, Sigma Aldrich) under UV light, rinsed twice with ultrapure water and treated for 10 min with 5% sodium thiosulfate and allowed to completely dry. The staining was evaluated by bright field optical microscopy, under 50× magnification.

### 2.4. RNA Extraction and Relative Quantification by Real-Time PCR

Total RNA extraction with TRIzol reagent was performed as described [37], for three time points; the moment of osteogenic induction (T1), after 7 days (T2) and 14 days (T3) of osteogenic induction. Total RNA was stored in TE buffer supplemented with 5 U/µL RNase inhibitor (EO0381, Thermo Fisher Scientific, Boston, MA, USA), under liquid nitrogen.

Single-stranded cDNA libraries for all hMSC, were obtained from 5–15 µg of total RNA for each sample, using a reverse transcription system (High-Capacity cDNA Reverse Transcription Kit, Applied Biosystems™, Waltham, MA, USA), according to manufacturer protocol. ARN and cDNA were quantified using a DeNovix NanoDrop spectrophotometer.

Gene expression levels of *ALPL*, *COL1A2*, *DCN*, *RUNX2*, *ELN* and *TGFΒ2* were measured using SYBR™ Green (SYBR) with confirmation of the original five signature genes using a TaqMan™ (Taqman) approach, for an inter-assay estimation of reproductibility. Both methods were performed using a Real-Time Quantum Studio™ 5 PCR thermocycler from Applied Biosystems (Thermo Fisher Scientific, Boston, MA, USA).

SYBR Green reaction mix for real-time PCR amplification/detection used a ready-mix (PowerUp™ SYBR™ Green Master Mix, Applied Biosystems), with 30 ng/µL specific cDNA and 2µM of each specific set of primers (Integrated DNA Technologies IDT, Inc, Coralville, Iowa, USA) (Table 1). Cycling was done with the sequence 50 °C for 2 min, 95 °C for 10 min, 40 × (95 °C 2 min, 51 °C 20 s, 72 °C 13 s), ended by a standard dissociation protocol for checking the specificity of the amplifications.

The TaqMan used a commercial ready-mix (TaqMan Fast Advanced Master Mix, 4444557, Thermo Fisher Scientific, Boston, MA, USA) with 10 ng/µL cDNA and primers/probes (TaqMan Gene Expression Custom Assays, Thermo Fisher Scientific, Boston, MA, USA) (Table 2). The PCR cycling protocol was 50 °C 2 min, 95 °C 2 min for polymerase system activation, and 40 × (95 °C 15 s, 60 °C 60 s).

For both SYBR Green and TaqMan methods, we tested three reference genes (*ACTB*, *GUSB* and *YWHAZ*). The PCR amplification efficiency was assessed for each target and reference gene using the calibration dilution curve and slope calculation from an equivalent mass ratio mixture of all cDNA samples in the library. For both methods we performed two separate experimental set-ups with triplicate samples for each testing parameter (cell line and induction time). Measurements from triplicate determination for both methods were statistically significant (*p* < 0.05) and the final fold-increase value per gene was the average between two parallel experiments. The relative gene expression for each sample was normalized to each of the three endogenous references using the 2^−ΔΔCt^ method and fold-increase for treated samples was calculated according to the Pfaffl algorithm [38].

The fold-increase gene expression for treated samples with a normalized function of two or three reference genes was calculated by determining the geometric average values for multiple internal control genes [39].

### 2.5. Median Absolute Dispersion (MAD) Analyses for Household Genes Selection

We used a Median Absolute Dispersion (MAD) analysis, in order to assess the best reference gene pair for the nine donor-specific hMSC populations. Deviation from the median for each possible combination of one to three candidate reference genes was calculated as the absolute value of differences between the gene fold-increase and median value of the dataset. For each reference gene combination, we analyzed the biomarker fold-increase gene induction values, comparing the original five signature genes with both SYBR Green and TaqMan methodology, and, subsequently, *TGFB2* with the SYBR Green method. A median absolute deviation score for each data set was calculated for one and two weeks of osteoinduction. The favoured reference gene and reference gene pair were those producing the smallest average MAD values among the nine donor-specific hMSC populations.

### 2.6. Hierarchical Cluster Analysis

Inter-donor similarity according to biomarker gene expression patterns in hBM-MSC and hAT-MSC treated with OM was determined using open-source versatile matrix visualization and analysis software Morpheus (Morpheus, https://software.broadinstitute.org/morpheus). Cluster algorithms for continuous variables were determined using the Euclidian distance and single linkage algorithm. Euclidian distance was appropriate for continuous variables sharing the same scale, taking the magnitude of changes into account to more comprehensively assimilate the gene expression data. The means for none-treated samples were set to zero, since our gene expression values represented a fold-increase in mRNA levels above a reference state determined by the control samples.

### 2.7. In Vivo Heterotopic Bone Formation

Adult immunodefficient mice NOD.CB17-Prkdc scid/J were used for in vivo testing of osteogenic potency. Mice handling was carried out in accordance with the EU Directive 2010/63/EU and national legislation (Law no.43/2014). All experimental procedures were previously approved by the Ethics Committee for Research of Vasile Goldis Western University of Arad (Approval no. 131/12.13.2018). Animals were housed in individually vented cages, with ad libitum access to food/water, standard conditions of temperature/relative humidity and a light/dark cycle of 12/12 h.

The MBCP+^®^ scaffold (Biomatlante, Vigneux de Bretagne, France), composed of a 20:80 ratio of hydroxyapatite (HA)/beta-tricalcium phosphate (β-TCP) by weight, was exposed for 2 h under UV light for sterilization. Serologic sterile syringes of 1 mL were cut at the apical part, filled with 70 mg of sterile material, and capped with sterile 1.5 mL centrifuge tubes. The scaffolds inside the syringes were washed twice using PBS (37 °C), for 10 min, followed by incubation at 37 °C, 5% CO_2_, with fresh PBS, overnight. The PBS was then replaced with MSCs suspension in αMEM. A total of 1.6 × 10^6^ hMSC from each donor were added in top of 40 mg (±5%, 40 granules) of 1–2 mm MBCP+^®^, granules. The syringes, loosely capped with inverted eppendorf tubes to allow gas exchange, were placed in the incubator in standard culture conditions for 24 h, then used for in vivo implantation. For the negative controls of each donor hMSCs, the scaffold was not seeded with cells and treated in the same conditions as positive samples. Visual inspection by inversed phase microscope of positive control samples of each donor-specific hMSC population showed adherent cells in the scaffolds.

The animals were anesthetized with ketamine/xylazine, and the dorsal skin of the mice was shaved and disinfected. Incisions of 1 cm were made in the upper and lower dorsal flank of each mouse. Blunt skin dissection formed a 3-cm-long pocket for subcutaneous graft implantation in the upper and lower dorsal flank of each mouse, as described [40].

Eight weeks after implantation, mice were euthanatized under anaesthesia and the explants were removed and collected for further histological analysis.

### 2.8. Histology and Immunohistochemistry

Ex vivo explants were fixed for 48 h in 4% paraformaldehyde, decalcified in 4% paraformaldehyde solution in PBS for one week, embedded in paraffin and cut in 5.0 μm thick sections. For morphological analysis of the new tissue, samples were stained with a Gomori’s trichrome kit (Leica Biosystems, Nussloch, Germany) to highlight collagen synthesis and osteoid deposition. Microscopic sections were analyzed with a BX43 microscope (Olympus Europa SE & Co, Hamburg, Germany).

Immunohistochemical staining was performed with recombinant rabbit monoclonal anti-mouse Runx-2 antibody (Abcam, ab192256, dilution 1:200), rabbit monoclonal anti-human osteopontin (Opn) (Abcam, ab63856, dilution 1:100), rabbit polyclonal anti-human TGF-β2 (Abcam, ab53778, dilution 1:100) and rabbit polyclonal anti-human decorin (DCN) IgG (Abcam, ab151988, dilution 1:200) primary antibodies. For visualization, Novocastra Peroxidase/DAB kit (Leica Biosystems, Nussloch, Germany) was utilized according to the manufacturers’ instructions.

## 3. Results

### 3.1. Establishment of hBM-MSC and hAT-MSC Expansion in Culture

**Figure 1:**
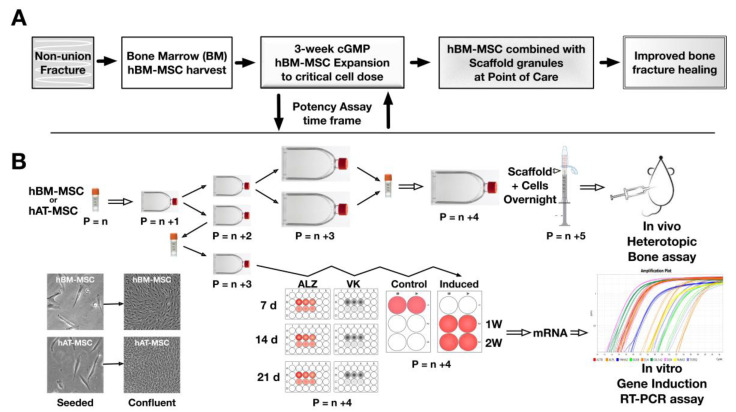
Schematic diagrams of: (**A**) A therapeutic approach for non-union bone fractures applying autologous bone marrow (BM) derived human multipotent stromal cells (hMSC) derived and expanded in culture for 3 weeks under current Good Manufacturing Practice (cGMP). Expanded cell cultures reaching a therapeutic dose were combined with osteogenic scaffold granules at point of care, with a view to promoting hBM-MSC osteogenic differentiation and improved bone fracture healing. (**B**) Our preclinical stage studies involved expansion of bone marrow derived (hBM-MSC) and adipose tissue derived (hAT-MSC) from frozen primary cell stocks, seeded in monolayer culture flasks, maintained in grow medium until near-confluence before serial passage into flasks. Since off-site in vivo experiments required frozen vial transport, congruence was maintained by introducing a cell freezing step before transferring cells to multi-well plates for treatment with osteogenic induction medium. Osteogenic differentiation was assessed by Alizarin Red S (ALZ) and Von Kossa (VK) staining to detect biomineralization at 7, 14 and 21 days, whilst parallel cultures were in osteogenic induction medium for 1 and 2 weeks before harvesting mRNA for quantified real-time PCR analysis of osteogenic signature gene induction relative to non-induced control cells.

hBM-MSC and hAT-MSC stored in cryotubes under liquid nitrogen at early passage (n), were carefully thawed from frozen vials and expanded in culture under cGMP-like growth conditions similar to corresponding protocols adopted for clinical trials (Figure 1A). Culture procedures for each donor-specific culture followed a specific protocol (Figure 1B) so that cells underwent only one freeze-thaw cycle and a near-equivalent number (n) of passage transfers (P) before concordant evaluation of in vitro osteogenic differentiation (P = n) or in vivo bone formation (P = n + 1). For all nine donor-specific hMSC populations, sufficient cells for experimentation were attained within 10 cumulative population doublings.

**Figure 2:**
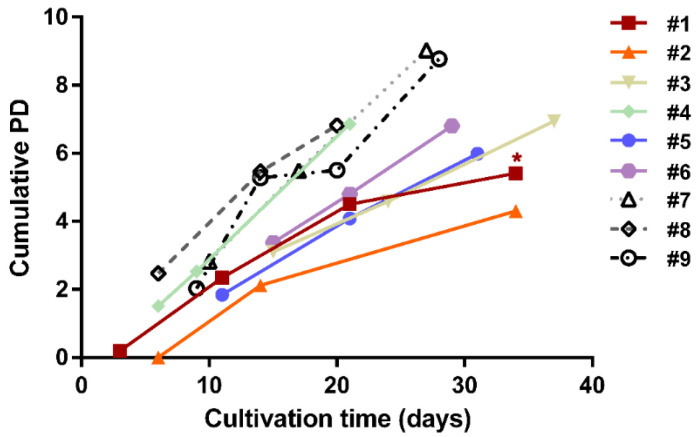
Growth curve of hBM-MSC and hAT-MSC expansion in culture showing cumulative population doubling (PD) between successive passages before experimental analysis. Donor #1 cells showed late-stage slow growth (* *p* < 0.05).

Given that a finite growth potential may impinge upon cell function, the growth rate was monitored for each donor-specific MSC population. For 8/10 donor-specific cultures, an exponential growth rate was maintained to the point of experimentation, with hAT-MSC from donors #7, #8, #9 showing doubling times of 3, 4.4 and 2.4 days, respectively (Figure 2).

Although the population doubling time for hBM-MSC from donor #2 slowed from an initial 2.1 days to 4.3 days, the cells remained competent bone formers (Table 3). However, donor #1 cells showed a markedly slowed growth rate, with population doubling times of 21 days at passage 7 and 34 days at passage 8, likely to reflect the onset of proliferative senescence (not tested). Consistent with hAT-MSC having greater proliferative potential than hBM-MSC [41] the median growth rate of our three hAT-MSC cultures at harvest was 3.0 days (range 2.4–4.4 days), whereas for the six hBM-MSC cultures it was 5.4 days (range 2.9–14.3 days). Nonetheless, for 5/6 cases, the previously reported bone-forming potential of the hBM-MSC was confirmed with the exception of donor #1, for which in vivo bone-forming potential was not determined (ND) (Table 3).

### 3.2. Osteogenic Medium Promptly Induced hBM-MSC and hAT-MSC Collagen Matrix Biomineralization

**Figure 3:**
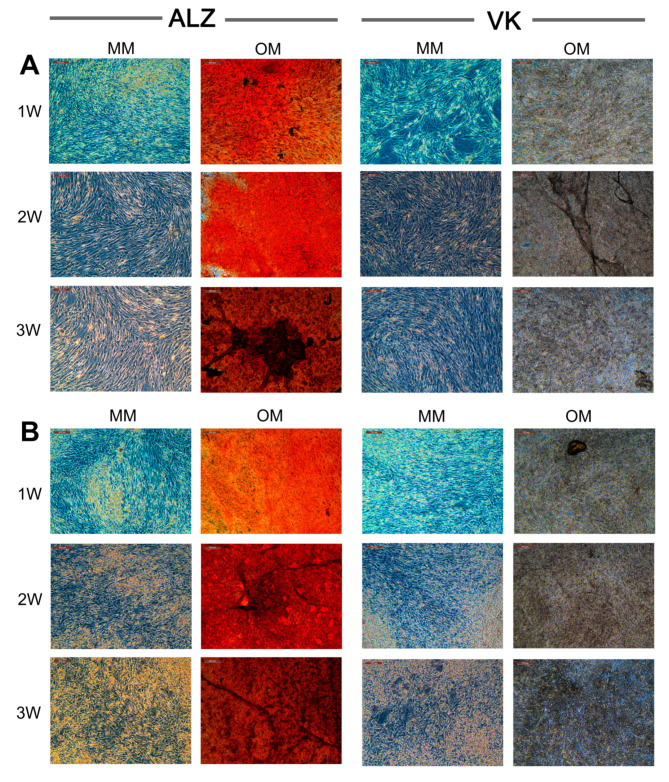
Representative photomicrographs of (**A**) hBM-MSC and (**B**) hAT-MSC grown in maintenance medium (MM) or treated with osteogenic medium (OM) to induce extracellular matrix biomineralization, detected using Alizarin Red S (ALZ) or Von Kossa (VK) stains at 1-, 2- and 3-week time points.

In agreement with the claimed efficacy of OsteoMAX-XF™, induction of a biomineralized extracellular collagen matrix shown by positive ALZ and VK staining, was consistently observed for all the hBM-MSC and hAT-MSC tested (Figure 3). For cells derived from donors #5 and #6 in particular, this was more robust that the mineralization previously observed for these hBM-MSC populations when using a proprietary osteogenic induction medium [18].

**Figure 4:**
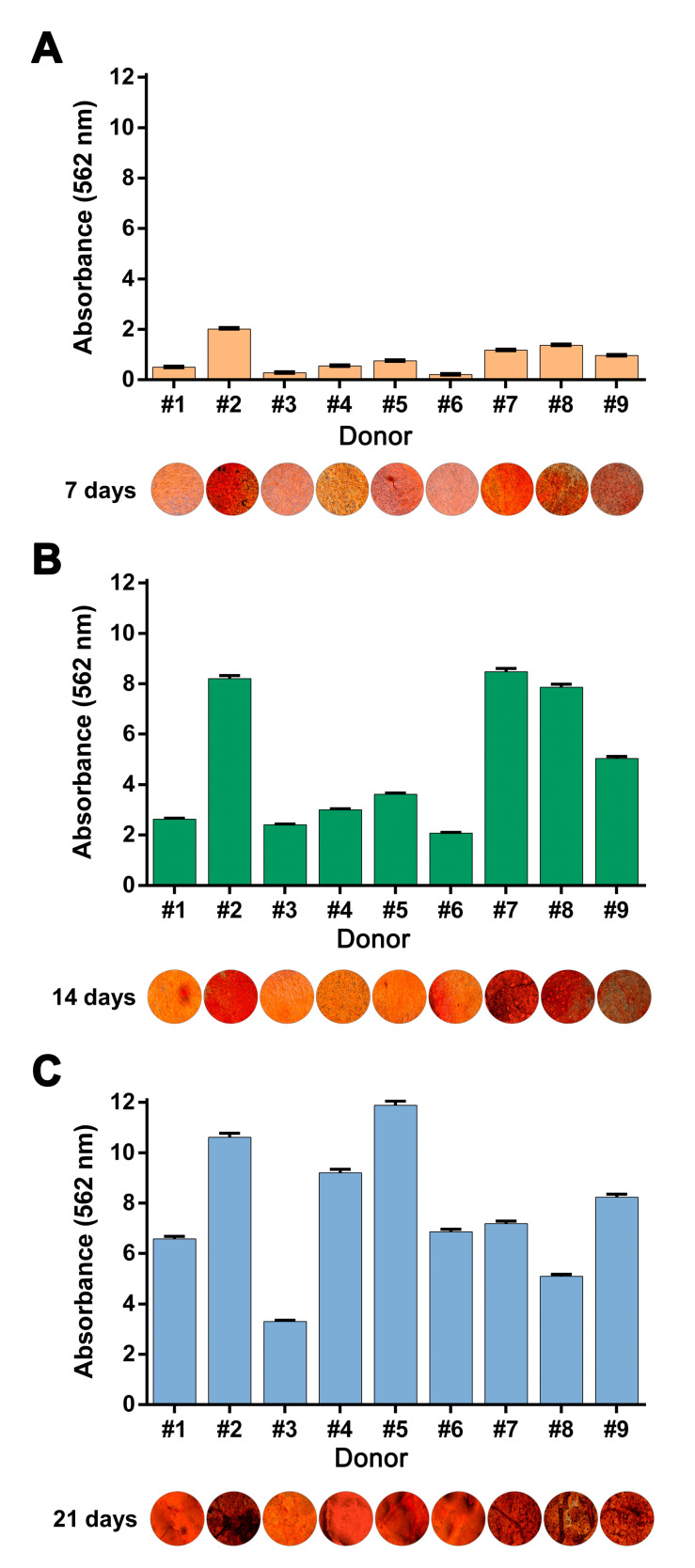
Inter-donor heterogeneity of quantified Alizarin red S staining of hMSC derived from donors #1–#9 treated with osteogenic medium for (**A**) 7, (**B**) 14 or (**C**) 21 days.

Quantification of mineralization detected by Alizarin Red S showed incremental accumulation of extracellular matrix mineralization over a three-week time course for both hBM-MSC and hAT-MSC (Figure 4).

### 3.3. The Real-Time PCR Reference Gene Pair of Lowest Median Absolute Dispersion (MAD) Was GUSB/YWAHZ

The osteogenic potency assay’s key quantitative parameter was the relative mRNA induction of signature gene biomarkers normalized to an internal reference control. Normalization was a principal concern for reliable quantitative comparisons because it controls for variations in extraction yield and efficacy of amplification, improving the comparison between different samples. Experimental validation of internal reference genes allowed more accurate reporting of the RNA concentration ratios of the genes of interest. We sought to determine the most stably expressed reference genes also having an abundance correlated to the total amounts of mRNA for the target genes. Consistent with previous reports of reliable reference genes when performing quantitative real-time PCR in hMSC undergoing differentiation [27], we observed lower average median absolute dispersion scores for *GUSB* (3.99) and *YWAHZ* (6.64) than for *ACTB* (10.75). Notably, *ACTB* was the least stable reference gene in a study that evaluated hAT-MSC in platelet lysate growth conditions [42]. Following recommendation for use of two internal control genes, we found the combination of *GUSB* and *YWAHZ* constituted the gene pair with lowest MAD when using SYBR Green methodology. This gene pair combination also provided the best representative average value when comparing the three gene pair combinations of *ACTB*/*GUSB* or *ACTB*/*YWAHZ* or *GUSB*/*YWAHZ* using TaqMan methodology (Table 4). Therefore, the *GUSB*/*YWAHZ* gene pair was chosen for donor-specific comparison of signature gene expression.

### 3.4. Inter-Donor Heterogeneity for Osteogenic Potency Signature Gene Induction in hBM-MSC and hAT-MSC

**Figure 5:**
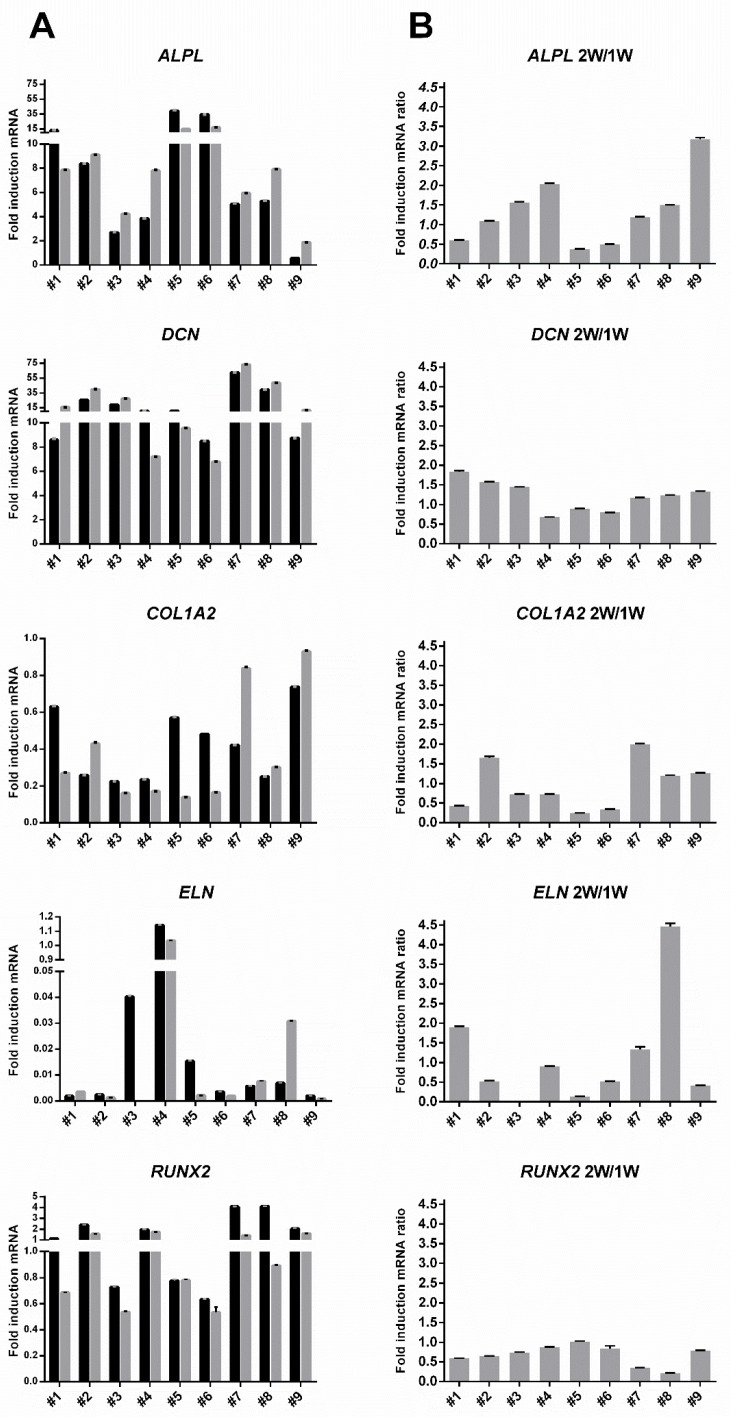
Inter-donor heterogeneity for fold increase in osteogenic signature gene induction against corresponding non-induced hMSC derived from donors #1–#9 detected by the SYBR Green method. Histograms of column (**A**) indicate the level of named gene upregulation in response to osteogenic medium treatment for 7 days (black bars) or 14 days (grey bars). Histograms of column (**B**) indicates the ratio between fold increase values at two weeks (2W) and one week (1W). Interpolation used GUSB/YWAHZ reference genes. Inter-assay variability is shown by error bars indicating the pooled coefficient of variation (*p* < 0.05).

With reassuring similarity to our previous study concerning osteogenic potency assay biomarkers using five signature genes, individual hBM-MSC and now also hAT-MSC populations showed striking donor-specific gene expression patterns for *ALPL*, *COL1A2*, *DCN*, *ELN* and *RUNX2* gene induction after treatment with osteogenic medium (Figure 5 column A). As previously reported, *ALPL* induction was most elevated in hBM-MSC from donors #5 and #6, and there was close parity with data obtained from cells differentiated for two weeks as opposed to just one week (Figure 5 column B). Among the similarities and differences in the cross-laboratory donor-specific clustering outcomes, hBM-MSC from poor-bone-forming donor #5 and outlier donor #6 [18] were still classified as distinct from the bone-forming donors. However, in contrast to previous analysis of these genes in hBM-MSC [18], there were no examples where a signature gene showed extreme induction levels (>100-fold) and the levels of induction of *COL1A2*, *ELN* and *RUNX2* were minimal. After 1 week of osteogenic induction, only *RUNX2* showed up to about 2-fold induction in hBM-MSC donors #2 and #4 and up to about 4-fold in hAT-MSC donors #7 and #8. More consistently, *ALPL* (typically ≥ 5-fold) and *DCN* (typically ≥ 10-fold) showed readily measurable levels of induction. These cross-laboratory differences might reflect the following: (i) use of different real-time PCR apparatus and formats (a 96-well as opposed to 24-well system), (ii) that the hBM-MSC were analyzed after they had undergone additional freeze-thaw cycles and passage in culture, (iii) different growth conditions from use of an alternative commercial platelet lysate and osteogenic induction formulation, and (iv) use of alternative real-time PCR reference genes; a *GUSB*/*YWAHZ* pair as opposed to *ACTB* alone.

### 3.5. TaqMan Real-Time PCR Confirmed Low Induction of COL1A2, ELN and RUNX2 Genes

**Figure 6:**
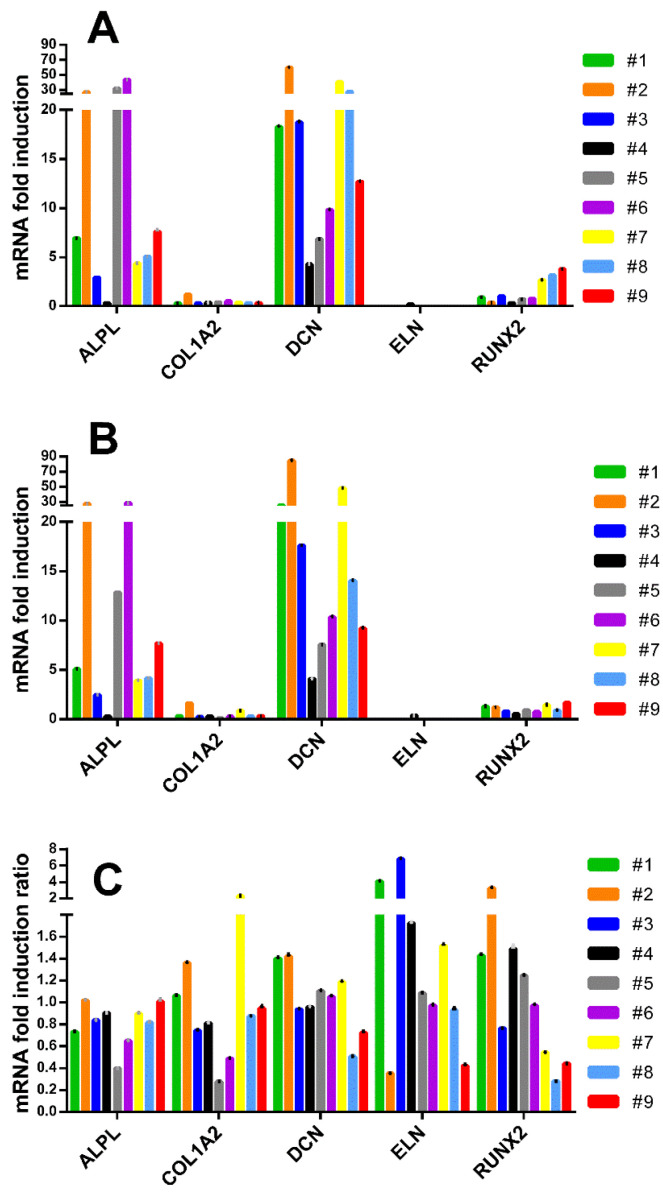
Inter-donor heterogeneity for fold increase in osteogenic signature gene induction against corresponding non-induced hMSC derived from donors #1–#9 detected by the TaqMan™ method measured at (**A**) 1 week and (**B**) 2 weeks. (**C**) Close parity for gene induction at the 1- and 2-week time points resulted in most gene induction ratios being close to one. Interpolation used GUSB/YWAHZ reference genes. Inter-assay variability is shown by error bars indicating the pooled coefficient of variation.

To test whether strikingly different overall results in signature gene induction levels compared to our previous studies did not simply reflect SYBR Green related technical discrepancies, we repeated the analysis using TaqMan real-time PCR technology (Figure 6).

Again, as for SYBR Green based PCR, TaqMan analysis indicated that induction of *ALPL* and *DCN* was notably greater than for the other signature genes *COL1A2*, *ELN* and *RUNX2*. Indeed the reproducibility with our SYBR Green data was very high, confirming previous reports that use of high performance primers and careful protocols rendered similar performance from both SYBR Green and TaqMan methods [43]. *COL1A2* and *ELN* were barely induced and *RUNX2* expression at 1 week was induced closer to 4-fold in hAT-MSC than hBM-MSC. Low induction of a signature gene did not necessarily indicate low constitutive levels of its expression, yet it has recently been observed that *COL1A2* gene silencing did not necessarily compromise murine embryo fibroblast in vitro mineralization or collagen matrix formation in vivo [44]. 

### 3.6. Cross-Laboratory Comparison of Potency Assay Cluster Analysis

The open access versatile matrix visualization software hosted by the Broad Institute, Cambridge, MA, US, provided a convenient platform for visualizing the inter-relationship of the different donors according to the gene expression profile of their derived hBM-MSC. Morpheus software cluster analysis of prior signature gene mRNA expression data produced a dendrogram diagram matching that previously obtained open source software Cluster 3.0 (ENCODE Project) [18].

**Figure 7:**
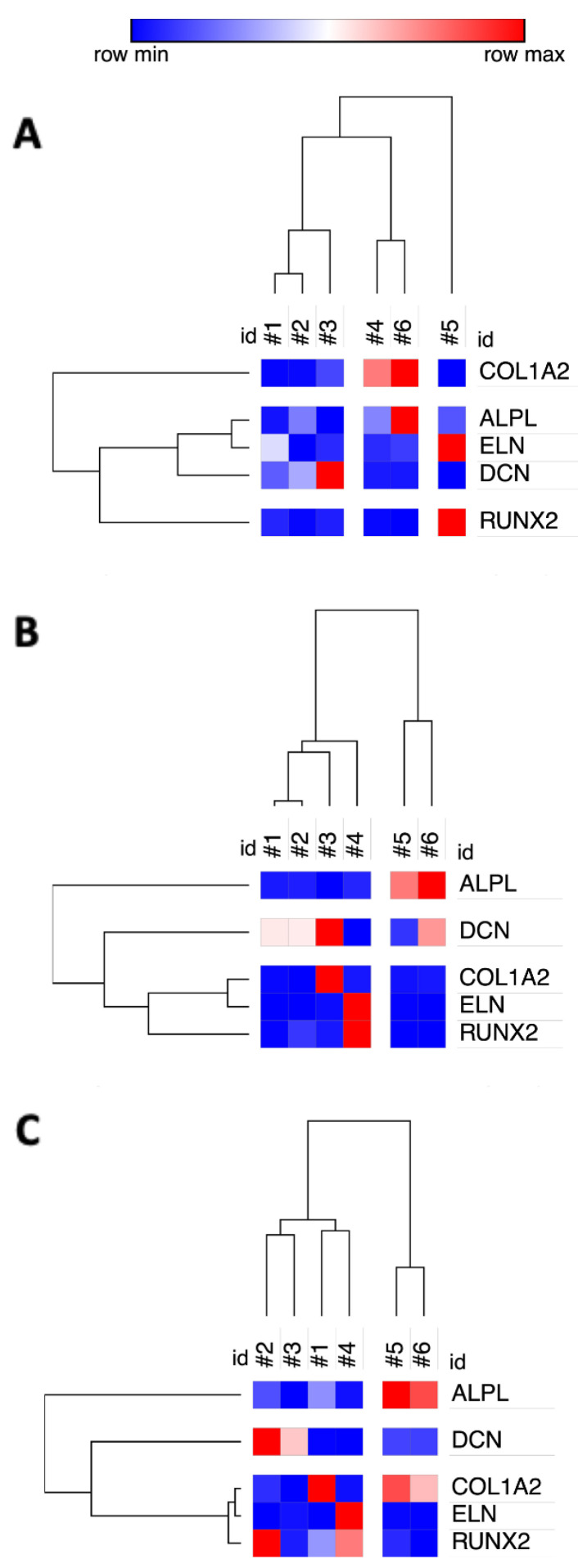
Cross-laboratory comparison of dendrograms from cluster analysis restricted to the five named osteogenic signature genes derived from (**A**) data from Murgia et al., 2016, using an *ACTB* reference gene, (**B**) our data using an *ACTB* reference gene or (**C**) our data using the *GUSB*/*YWAHZ* reference gene pair.

Given differences in experimental parameters and gene induction patterns from the previous comparative study [18], it was not surprising that the current derived cluster analysis was not identical (Figure 7A). Nonetheless, directly comparing the results retaining the same reference gene *ACTB* and some salient consistent features remained. The bone-forming hBM-MSC group constituted by donors #1, #2 and #3 remained clustered (Figure 7B), yet this group now also became associated with the non-bone-forming donor #4 cells. In the previous study, the non-bone-forming donor #4 cells were associated with donor #6 cells that had an outlier phenotype of being poor for matrix mineralization in vitro, yet forming bone in vivo. In our study, donor #6 cells were grouped with the poor-bone-forming donor #5 hBM-MSC, largely on the basis of both having distinctively high induction of *ALPL*. A main difference between the two studies was the different grouping of the signature genes, previously *COL1A2* had shown high levels of induction, whereas now *COL1A2* was clustered with *ELN* and *RUNX2* reflecting relatively low induction levels, observed when cluster analysis was performed from either SYBR Green or Taqman data (Figure 7C).

### 3.7. TGFB2 Gene Induction Was Distinct for hBM-MSC Versus hAT-MSC

**Figure 8:**
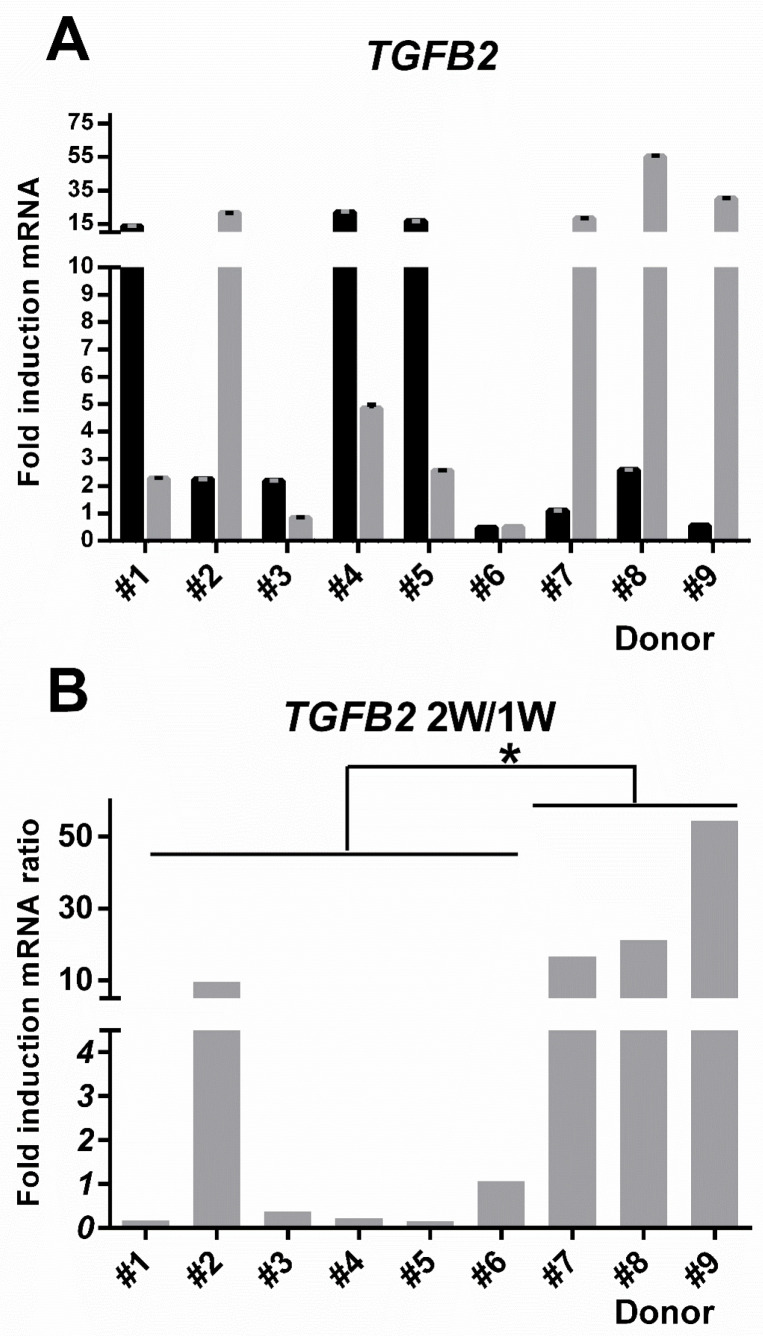
Inter-donor heterogeneity for fold increase in *TGFB2* gene induction versus corresponding non-induced hMSC control cells derived from donors #1–#9. (**A**) Gene upregulation in response to osteogenic medium treatment for one week (1 W) (black bars) or two weeks (2 W) (grey bars). Error bars indicate the pooled coefficient of variation. (**B**) The fold-induction ratio for values at one or two weeks post induction. Interpolation used the *GUSB*/*YWAHZ* reference gene pair. Independent t-test between hBM-MSC and hAT-MSC groups * *p* < 0.05.

With concern that under the new, more standardized assay conditions, the performance of the potency assay might be compromised by low levels of COL1A2 and ELN gene induction, we sought to test the suitability of *TGFB2* gene induction. More aligned with the well-expressed biomarkers *ALPL* and *DCN*, the levels of *TGFB2* induction at one week reached over 10-fold for half of the donor-specific hBM-MSC (Figure 8A). Moreover, the parity for gene induction at one week and two weeks was now different to all the previous potency assay signature genes, because there was clear discrimination between hBM-MSC and hAT-MSC (Figure 8B). The chronology of *TGFB2* gene induction for hAT-MSC was distinctly low at week one, but much higher at week two, an incremental induction that was less prevalent among the hBM-MSC and only seen to a lesser extent in hBM-MSC derived from donor #2. Notably, unlike *ALPL* or *DCN*, the overall pattern for *TGFB2* induction was more transient, a characteristic of in vivo relevance, noted in studies of TGF-β1 eliciting a pro-chondrogenic response to BMPs in early limb skeletogenesis [45].

### 3.8. Donor-Dependent Bone Formation by hBM-MSC and hAT-MSC with Distinct Decorin and TGF-β2 Immunohistology

**Figure 9:**
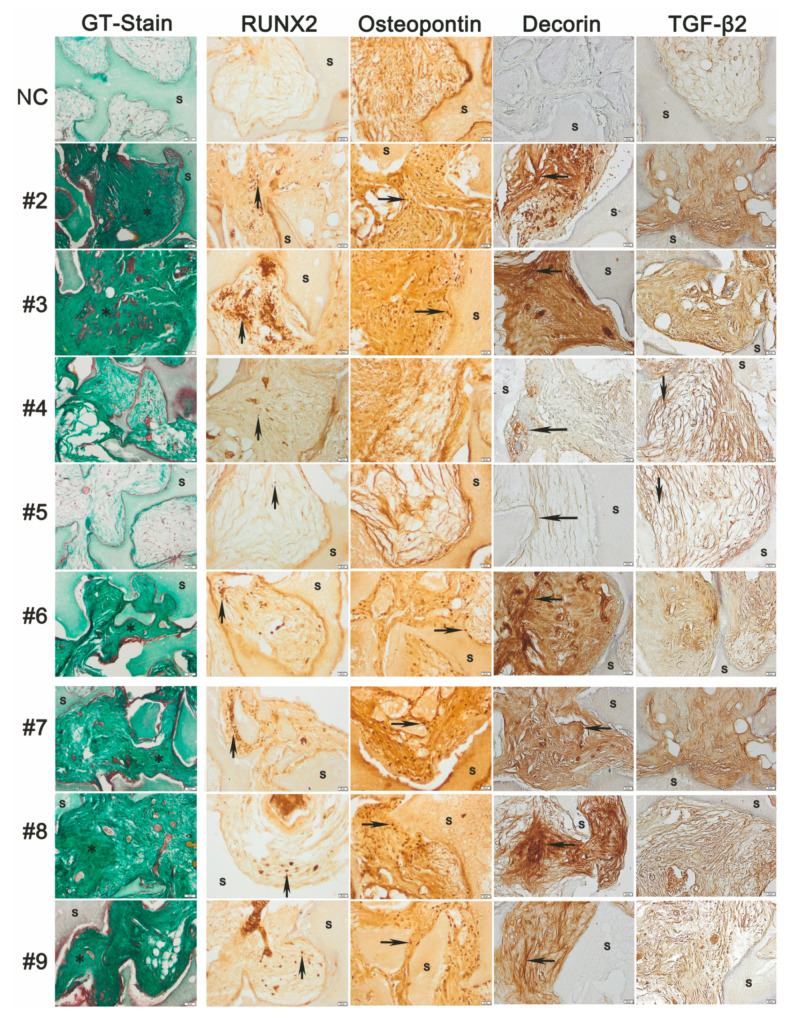
Histological analysis of inter-donor heterogeneity for in vivo osteogenic differentiation of hMSC derived from hBM-MSC donors #2–#6 and hAT-MSC donors #7–#9 implanted for 8 weeks with HA/βTCP scaffold (s) stained for osteoid/bone (*) with GT (Gomori’s trichrome stain). Negative control (NC) sections involved scaffold implants without human cells. Peroxidase immunohistochemical staining (brown) detected cells positive for the nuclear transcription factor RUNX2 (up arrow), Osteopontin (right arrow), human-specific decorin that densely stained cells from bone-forming donors (left arrow) and TGF-β2 protein that was diffusely present in the extracellular matrix of bone-forming cells and more distinctly stained the matrix of non-bone-forming (down arrow).

Histological analysis of heterotopic implants of hMSC from donors #2–#9 with osteoconductive scaffolds revealed inter-donor heterogeneity in vivo (Figure 9).

Notably, the hBM-MSC potential to form bone-like osteoid was consistent with previous studies [18]; here, we extended analysis of bone-forming potential per se to include immunohistochemical detection of Runx2 and Decorin as well as the extracellular matrix bone remodelling protein osteopontin and the novel candidate potency biomarker TGF-β2. Meeting the aim that the potency assay in vitro should have relevance for in vivo bone formation, we observed close correlation between the staining patterns of decorin and the propensity for the hBM-MSC to form bone. For sections belonging to donors #4 and #5, whose hBM-MSC did not form heterotopic bone, staining with the human-specific anti-decorin antibody was much weaker than for all other hBM-MSC and hAT-MSC that had a strongly positive staining pattern consistent with a dense osteoid matrix indicative of bone-forming potential. Moreover, the TGF-β2 staining pattern in sections from donors #4 and #5 displayed a more reticular pattern in contrast to a more diffuse staining pattern seen in the other hBM-MSC sections. Together, the histological staining patterns for the decorin and TGF-β2 proteins distinguished the sections from donors #4 and #5 as distinct; indeed, this correlated perfectly with the bone-forming potential of the hBM-MSC.

### 3.9. Inversely Correlated TGFB2 and DCN Gene Induction and Prospective Recognition of hMSC Bone-Forming Potential In Vivo

**Figure 10:**
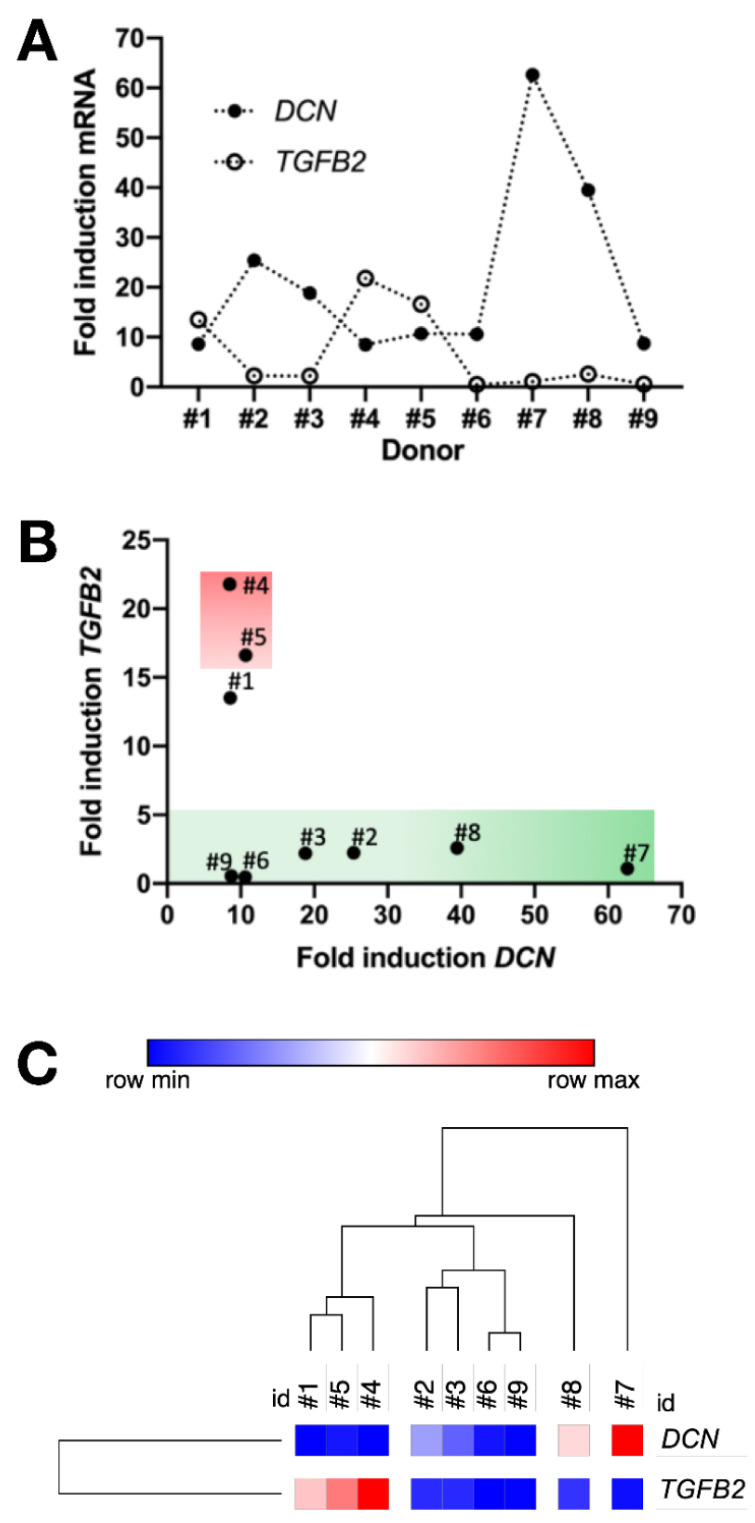
Prospective identification of osteogenic potency derived from (**A**) the inter-donor heterogeneity for fold-induction of gene expression for *DCN* and *TGFB2* after one week of osteoinduction, tracing an inverse correlation expression pattern across the hMSC derived from donors #1 to #9, allowing (**B**) a correlative plot between these two genes discriminating non-bone-forming hMSC (red zone) versus bone-forming hMSC (green zone). (**C**) Dendrogram of Euclidian cluster analysis for all donors using gene induction data from *DCN* and *TGFB2*.

Tracing induction of *DCN* and *TGFB2* genes one week after cell treatment with osteogenic medium across the different hBM-MSC samples revealed an inverse relationship (Figure 10A) that was particularly evident for donors #1 to #5 (Spearman correlation, *p* < 0.05). A less precise correlation was observed for the hAT-MSC; nonetheless, it could be maintained that if decorin induction was relatively high, TGFB2 induction was very low.

Strikingly, the plot of fold-induction at one week for *TGFB2* mRNA versus that for *DCN* mRNA revealed a donor-specific distribution pattern on the scatter graph that correlated extremely well with bone-forming potential, such that a “green zone” for donors displaying good bone-forming potential was clearly distinct to a “red zone” for hBM-MSC from donors #4 and #5 that did not form bone (Figure 10B).

The donor-specific clustering obtained using just the *DCN* and *TGFB2* induction data (Figure 10C) highlighted that the non-bone-forming hBM-MSC were distinct from the bone-forming hBM-MSC with further discrimination of hAT-MSC derived from donors #7 and #8.

## 4. Discussion

Heterogeneity among the cells of multipotent differentiation potential derived from connective tissues, generally referred to as “mesenchymal stromal/stem cells” [46], a contested nomenclature [47], can reflect choices of tissue source, detection method, isolation protocols, purification [48] and/or unique characteristics pertaining to the donor [49]. The heterogeneity in bone-forming potential, most readily demonstrable at the clonal level, can remain apparent in donor-specific hMSC cultures where pragmatic cell isolation using plastic culture adherence fails to discriminate functionality among morphologically similar fibroblastic cell types from various connective tissues [50]. Challenges presented by hMSC heterogeneity for clinical development [51], beckon assays measuring a quantifiable functional phenotype forecasting donor-dependent bone-forming potential for accurate control of therapeutic application.

Prior experimental analysis of 18 potential osteogenic biomarkers in six donor-specific hBM-MSC populations induced osteogenically for one week, resulted in selection of five significantly induced genes. These “signature genes” provided a collective expression pattern that helped cluster hBM-MSC in accordance with an in vivo heterotopic bone formation assay [18]. The eligibility of these functional biomarkers was supported by numerous studies: Tissue-nonspecific alkaline phosphatase (ALPL/TNAP), a homotetrameric glycophosphatidylinositol-anchored ectoenzyme, located to osteoblast cell surfaces at skeletal tissue calcification sites. *ALPL* was induced early during stem cell transit towards an osteoblast phenotype and could regulate osteogenic differentiation either by influencing *RUNX2* gene expression or by increasing the extracellular inorganic phosphate (Pi) concentration by hydrolysis of inorganic pyrophosphate (PPi) [52,53]. Often used to assess osteogenic differentiation, though not always correlated with bone formation [16], *ALPL* was induced in about 70% of independently examined hBM-MSC [54]. The *COLIA2* gene product combined with *COL1A1*-derived procollagen forms the collagen type 1 heterotrimer fibrils, (two alpha1(I) chains and one alpha2(I) chain), the principal matrix component of bone. Mutations in either procollagen gene were found in osteogenesis imperfecta patients, suggesting a fundamental role in bone development [55]. Nonetheless, in a murine *COL1A2* silenced model, osteoblasts could still form mineralizable collagen alpha1(I) homotrimers [44]. Decorin (DCN), one of several secreted glycosaminoglycan (GAG) noncollagenous proteins of bone, was characterized in bone as a leucine-rich repeat core protein covalently attached to chondroitin sulphate as opposed to a dermatan sulphate chain in soft connective tissues [56]. It binds and “decorates” collagen fibrils, dramatically influencing collagen fibril assembly and mechanical properties. Electron microscopy indicates decorin is decreased in more calcified fused collagen fibrils [57]. Elastin (ELN) microfibrils make essential cross-linked protein structures in the ECM that can increase bone remodelling in a defect area [49,50] and also modulate TGF-β availability [58]. Notably, hBM-MSC, selected for the CD271^+^ biomarker proposed to indicate functional competence, had a molecular signature involving enhanced expression of ECM proteins, including elastin [59]. RUNX2 a key bone development transcription factor essential for osteoblast differentiation, induces progenitor cell commitment to osteoblast lineage cells [60], regulating their proliferation and differentiation via reciprocal regulation with the hedgehog signalling pathway. *RUNX2* DNA-binding sequences occupy the promoters of almost all bone-specific genes [61]. Yet, *RUNX2* expression is not exclusively confined to differentiating cells and whether its induction predicted osteogenic potency in human cells was debatable [16].

Concerted effort from different laboratories to support qualification of biomarkers has been recommended [62]. We aimed to develop a robust, broadly applicable assay, suitable for biosensor development, to promote consistency across multi-centric trials, providing improved uniformity and scalability. Recapitulating a previously described potency assay [18], we paid special attention to stability, use of scalable reagents and scope for applicability to MSC from adipose as well as bone marrow tissue sources.

Cell proliferation and differentiation results supported the view that cryopreserved hMSC regained functional potency soon after acclimatization [63] and that platelet lysate provided excellent supportive growth medium for the culture of both hBM-MSC [64] and AT-MSC [65], confirming an expectation that hAT-MSC would grow faster than hBM-MSC [25]. With one possible exception (hBM-MSC from donor #1), the primary hMSC underwent four to eight cumulative population doublings without morphological changes or a growth rate plateau suggesting the onset of senescence [66]. Donor-specific differences in bone-forming potential in vivo, consistent with earlier studies, further verified that the cells were suitable for testing the quantitative potency assay.

We used the induction reagent OsteoMax-XF™, originally derived by screening about 3500 combinations of serum and xeno-free medium to obtain an efficient, fully defined reproducible medium for scalable hMSC osteoblast differentiation assessed by ALZ staining. Favorably compared to other osteogenic media, OsteoMax-XF™ induced rapid ECM calcification in dental pulp stem cells [29] and even human primary fibroblasts [28]. Notably, donor #6 hBM-MSC, previously staining ALZ negative unless the osteogenic induction medium was modified [18], were here ALZ positive.

Real-time PCR, the principal quantitative assessment method for our osteogenic potency assay, provided advantages of sensitivity, large dynamic range, accurate quantification and potential for automation. Heeding concern that *ACTB* may be inadequate for normalization of hMSC differentiation studies [27,67], we confirmed reports that GUSB [68] and YWHAZ [69] were more stable reference genes. We actually found *ACTB* to be acceptably stable for hBM-MSC but less consistent for hAT-MSC. We did not agree that GUSB was a poor reference gene for hAT-MSC [70], perhaps reflecting our use of FBS-free culture media. The MIQE guidelines discouraged large discrepancies in transcript abundance between reference and target genes and recommend more than one reference gene [71]. *GUSB*, the gene with the smallest average median absolute dispersion, had an average threshold cycle level suitable for measurement of *ALPL*, *DCN* and *TGFB2* levels of transcription, whereas *YWAHZ* mRNA levels matched the more weakly expressed *RUNX2* gene. Thus, the *GUSB*/*YWAHZ* reference gene pair provided a transcript abundance range suited to the target genes.

Numerous laboratories addressing therapeutic translation of heterogeneous, nonclonal mixtures of hMSC have yet to derive a comprehensive unequivocal quantitative potency assay that predicts bone-forming potential [72,73,74]. Considerable variability for hMSC biomarker mRNA levels between biological samples was a common observation [75]. Since microenvironmental factors readily influence readout variability, it was not surprising that we did not obtain an identical clustering outcome when using our modified version of the previously reported potency assay. Nonetheless, salient aspects were preserved. Firstly, the donor-specific hBM-MSC heterogeneity regarding bone-forming potential in the heterotopic immunocompromised mouse model remained consistent. Consistent donor-specific heterogeneity persisted despite opportunity for phenotypic drift during in vitro expansion of the hBM-MSC, as reported during prolonged growth of hMSC from bone marrow [76] and other tissue sources [77]. We confirmed prompt induction of osteogenic differentiation, with cell aggregation and ALZ-positive calcified ECM after just one week of treatment with osteogenic medium. This likely reflected use of platelet lysate instead of fetal bovine serum in the maintenance medium [78] and was also consistent with the reported effectiveness of OsteoMax-XF™.

Variability in signature gene mRNA induction allowed cluster analysis to discriminate a non-bone-forming hBM-MSC population as clearly distinct from the bone-forming hBM-MSC populations. In contrast to previous data, our *COL1A2* induction levels were lower than those previously reported [16,18]. Nonetheless, others found that collagen I barely showed any induction in hBM-MSC-TERT+Bone cells [16] or expressed early at day 5 in murine BM-MSC was down-regulated through osteogenic differentiation, although still transcribed at a suitable level for protein synthesis [79]. *DCN* induction may not be a feature of osteogenic differentiation in every context [80] yet we provide the third cross-laboratory example of its robust induction in bone-forming hBM-MSC grown in monolayer [16,18] or 3D culture conditions [68]. Our relatively low *ELN* induction levels may reflect inhibitory components of the proprietary induction medium; for instance, bFGF can down-regulate ELN expression [81], or high levels of inorganic phosphate in the ECM [82] from strong *ALPL* induction. Nonetheless, consistent low *ELN* induction levels among all our hMSC indicated attainment of advantageously stable potency assay conditions. Appropriate for a key osteogenic transcription factor [83] RUNX2 was induced, although only weakly. This was consistent with reports that primary human osteoblastic differentiation was associated primarily with post-translational changes in RUNX2 protein activity rather than a significant change in mRNA or protein levels [84].

Under our particular potency assay conditions, several biomarkers (especially *ELN*, *COL1A2*) showed relatively low (typically < 2-fold) induction levels. To strengthen the assay, we sought a further biomarker and focused on the TGF-β pathway, of notable interactive relevance to the set of five signature genes [18]. Of the three members of the TGF-β cytokine family (TGF-β 1–3), only *TGFB2* gene deletion induced defects in endochondral bone formation [85] supporting gene expression profile studies indicating a specific role in commitment to osteogenesis [32]. Though similar, mRNA and protein expression of TGF-B2 showed distinct patterns of expression to TGF-β1 in human bone samples [86]. We were encouraged to find that *TGFB2* induction was very detectable in hBM-MSC after one week of osteogenic treatment and intrigued that this was not the case for hAD-MSC. Extending the assay revealed that, in compensation, *TGFB2* was strongly induced in hAD-MSC, at a later two-week time point, consistent with the concept that hAD-MSC differentiation in vitro may lag that of hBM-MSC [87]. Despite many similarities, tissue-origin-specific differences between hBM-MSC and hAT-MSC may be revealed at the mRNA level [22,88], a point worthy of further investigation and consideration for the timing of tailored potency assay measurements.

Independent evidence supported the view that the gene expression data we obtained one week post osteogenic induction had good in vivo relevance. RNA expressed for diverse time points during in vitro osteogenic differentiation of ten hBM-MSC donors between passages 2 and 5 has been compared with primary cultures of normal osteoblasts isolated from hipbone, using microarray analysis of almost 30,000 genes. It was concluded that hBM-MSC on day 7 of their osteogenic differentiation were most similar to the non-mineralized primary human osteoblasts [89]. The authors highlighted that in vitro analysis of the osteogenic differentiation pathway demonstrated three developmental phases of proliferation, matrix maturation and mineralization with activation of the SMAD signalling pathways by members of the bone maturation protein (BMP) family and TGFB2.

Across the hMSC samples, we observed an apparent inverse correlation between the induction of *TGFB2* and *DCN*. Notably, among a wide network of protein interactions [90], decorin can interact directly with TGF-β proteins [91] and, when immobilized on collagen fibrils, decorin modulated TGF-β availability and activity [92]. Dynamic decorin and TGF-β inhibitory interactions modulated extracellular matrix organization significantly [93] and influenced cell migration [94]. All bone-forming hMSC showed *TGFB2* and *DCN* mRNA induction at one week within ≈5- and ≈75-fold, respectively, in contrast to relatively high *TGFB2* induction (>15-fold) and relatively low *DCN* induction (<15-fold) in non-bone-forming hBM-MSC populations from donors #4 and #5. The latter matched relatively low decorin detection in the histological sections the heterotopic implants belonging to donors #4 and #5. Conversely, relatively high *TGFB2* induction in vitro did not directly correspond to high TGF-β2 detection in histological sections. This apparent paradox may likely reflect complex post-trancriptional regulation of TGFB2 protein expression, including a sequestrating interaction with decorin [95]. Consistent complexity was found in a transgenic mouse model of osteoporosis; TGF-β2 functioned as a local positive regulator of bone remodeling, yet osteoblast-specific overexpression of this protein resulted in progressive bone loss [96].

The hBM-MSC derived from donor #1 demonstrated a unique relationship between *TGFB2* induction at one week and bone formation. Previous studies indicated the cells formed bone, yet here they showed relatively high *TGFB2* expression. Compared to the other hMSC samples, the growth curve for hBM-MSC donor #1 cells indicated the slowest growth rate before implantation, likely reflecting early onset of asynchronous replicative senescence associated with functional attrition [97,98]. Notably, TGF-β2 [99] but not TGF-β1 [100] could accelerate hBM-MSC senescence, a phenotype also imparted by TGF-β 2 in vivo during inner ear development [101]. In vitro senescence was not the focus of our study and these observations need further experimental verification. Nonetheless, senescence biomarkers are of interest for quality control of therapeutic cell preparation [102] and may complement the osteogenic biomarkers presented here [103].

We advance previous potency assay studies with confirmation that measurement of bone extracellular matrix metabolism in hMSC can provide strong deterministic indications regarding bone-forming potential. Unlike an orthotopic microenvironment, the subcutaneous murine model offered a relatively consistent and stringent donor-specific surrogate test of bone-forming potential [104] lacking stimulation from nearby bone cytokines and cell-cell interaction with host bone-forming cells. Despite a limited relevance of small animal studies to the human therapeutic context [105] the assay provided controlled in vivo experimental bone formation. Potency assays can be considered multiparametric “works in progress,” to be continuously updated and improved according to advances in protocols and multifaceted target knowledge [106]. Co-regulated gene indexes are likely to prove advantageous for future studies testing further novel osteogenic potency assay biomarkers. Genome-wide weighted gene Pearson correlation network analysis between pairs of genes has revealed highly-correlated novel biomarkers with consistent profiles across samples, relevant to early phase hMSC differentiation [107]. A novel genomic biomarker, characterized by loss of the gene-encoding glutathione S-transferase theta 1 (GSTT1) correlated with enhanced hMSC culturability [108]. Together with further enhanced culture methods [109], such potency biomarkers provide excellent prospects for improving the broad applicability of hMSC therapeutics.

*TGFB2* may be considered fully eligible as an osteogenic potency signature gene biomarker of high biological relevance to bone formation. Future studies may be proposed to explore whether it can also be indicative of hMSC growth potential. Under carefully standardized assay conditions, its mRNA induction could be readily quantified in both hBM-MSC and hAD-MSC and, combined with *DCN* induction, *TGFB2* discriminated hMSC according to their prospective bone-forming potential. Analysis of decorin and TGF-β2 protein expression in heterotopic bone implants also revealed distinct histomorphological patterns in non-bone-forming explants. Measurement of *TGFB2* mRNA induction enhanced the osteogenic potency assay’s discriminatory power for prompt prospective identification of non-bone-forming hMSC populations to improve regenerative medicine effectiveness.

## Figures and Tables

**Table 1:** Primer sequences for SYBR Green real-time PCR.

Gene Name	Primer Sequence	Amplified Length (bp)
Alkaline phosphatase (*ALPL*)	5’-GATGTGGAGTATGAGAGTGACG-3’ (sense) 5’-GGTCAAGGGTCAGGAGTTC-3’ (antisense)	142
Collagen type I, alpha 1 (*COL1A2*)	5’-AGGACAAGAAACACGTCTGG-3’ (sense) 5’-GGTGATGTTCTGAGAGGCATAG-3’ (antisense)	146
Decorin (*DCN*)	5’-AAAATGCCCAAAACTCTTCAGG-3’ (sense) 5’-GCCCCATTTTCAATTCCTGAG-3’ (antisense)	146
Elastin (*ELN*)	5’-CCTGGCTTCGGATTGTCTC-3’ (sense) 5’-CAAAGGGTTTACATTCTCCACC-3’ (antisense)	148
Runt related transcription factor 2 (*RUNX2*)	5’-TTCACCTTGACCATAACCGTC-3’ (sense) 5’-GGCGGTCAGAGAACAAACTAG-3’ (antisense)	148
Transforming growth factor beta 2 (*TGFB2*)	5’-CAAAATAGACATGCCGCCCTTC-3’ (sense) 5’-GAAGGGCGGCATGTCTATTTTG-3’ (antisense)	150
Actin beta (*ACTB*)	5’-ACCTTCTACAATGAGCTGCG-3’ (sense) 5’-CCTGGATAGCAACGTACATGG-3’ (antisense)	148
Glucuronidase beta (*GUSB*)	5’-CCAAGGGTTACTTTGTCCAGA-3’ (sense) 5’-TAATTCACCAGCCCACTGTC-3’ (antisense)	151
Tyrosine 3 monooxygenase/tryptophan 5-monooxygenase activation protein zeta (*YWHAZ*)	5’-TGACAAGAAAGGGATTGTCGAT-3’ (sense) 5’-TCTGGGGAGTTCAGAATCTCAT-3’ (antisense)	150

**Table 2:** TaqMan Real-Time qPCR Gene expression assays details.

Gene Name	Protein Function/Gene Group	TaqMan Assay
Alkaline phosphatase (*ALPL*)	Phosphatase (bone marker, gene of interest)	Hs01029144_m1
Collagen type I, alpha 1 (*COL1A2*)	Extracellular matrix structural protein (bone marker, gene of interest)	Hs01028956_m1
Decorin (*DCN*)	Collagen associated proteoglycan (bone marker, gene of interest)	Hs00370385_m1
Elastin (*ELN*)	Extracellular matrix structural protein (bone marker, gene of interest)	Hs00355783_m1
Runt related transcription factor 2 (*RUNX2*)	Transcription factor (bone marker, gene of interest)	Hs01047973_m1
Actin beta (*ACTB*)	Structural protein (housekeeping gene/control)	Hs99999903_m1
Glucuronidase beta (*GUSB*)	Lysosomal enzyme (housekeeping gene/control)	Hs00939627_m1
Tyrosine 3-monooxygenase/tryptophan 5-monooxygenase activation protein zeta (*YWHAZ*)	Signalling pathway protein (housekeeping gene/control)	Hs00237047_m1

**Table 3:** Overview of the number of hMSC passages in vitro and osteogenesis in vivo.

Donor	MSC Source	Passage of Cryopreserved hMSC (*n*)	Passage Number for In Vitro mRNA Harvest	Passage Number before In Vivo Heterotopic Implantation	Previously Quantified In Vivo Heterotopic Osteoid/Bone (%) ([18])	Bone Formation In Vivo (Present Study)
#1	BM	5	8	9	15.3	ND †
#2	BM	4	7	8	18.8	yes
#3	BM	4	7	8	18.1	yes
#4	BM	3	6	7	0.1	no
#5	BM	1	5	6	5.4	no
#6	BM	3	6	7	15.3	yes
#7	AT	1	4	5	ND	yes
#8	AT	5	8	9	ND	yes
#9	AT	4	7	8	ND	yes

† Not Determined.

**Table 4:** Comparative Median Absolute Dispersion (MAD) analysis of reference genes *ACTB*, *GUSB*, *YWAHZ*.

SYBR	MAD 1W	A/G/Y	A/G	A/Y	G/Y	A	G	Y
	*ALPL*	4.546	4.751	6.007	6.007	7.184	1.508	3.412
	*COL1A2*	6.944	6.854	6.778	6.973	6.703	6.997	7.127
	*DCN*	13.825	9.343	14.523	3.284	31.250	2.792	12.863
	*ELN*	7.391	7.392	7.390	7.389	5.746	5.749	6.934
	*RUNX2*	5.198	5.661	5.146	5.406	9.837	1.759	4.441
	*TGFΒ2*	6.364	6.508	6.438	6.299	3.754	5.125	5.087
	AVERAGE MAD	7.378	6.752	7.714	5.893	10.746	3.988	6.644
	MAD 2W	A/G/Y	A/G	A/Y	G/Y	A	G	Y
	*ALPL*	5.264	8.523	3.351	4.112	5.388	0.828	4.661
	*COL1A2*	11.575	11.623	11.464	11.641	11.167	11.383	11.640
	*DCN*	21.285	9.018	22.687	5.132	29.062	4.576	15.944
	*ELN*	11.908	11.908	11.906	11.909	9.490	9.495	11.452
	*RUNX2*	10.717	11.065	10.069	11.020	7.547	7.697	10.116
	*TGFΒ2*	8.966	9.050	10.541	9.633	8.134	7.261	10.001
	AVERAGE MAD	11.619	10.198	11.670	8.908	11.798	6.874	10.636
**TaqMan**	**MAD 1W**	**A/G/Y**	**A/G**	**A/Y**	**G/Y**	**A**	**G**	**Y**
	*ALPL*	2.879	3.553	3.553	3.553	5.135	4.113	3.899
	*COL1A2*	5.530	5.546	5.546	5.598	5.301	5.684	5.490
	*DCN*	13.710	11.984	11.984	12.322	20.311	6.782	19.807
	*ELN*	5.961	5.962	5.962	5.962	5.963	5.964	5.960
	*RUNX2*	4.564	4.616	4.616	5.054	4.276	5.355	4.765
	AVERAGE MAD	6.529	6.332	6.332	6.498	8.197	5.580	7.984
	MAD 2W	A/G/Y	A/G	A/Y	G/Y	A	G	Y
	*ALPL*	3.479	2.817	3.192	4.249	5.986	4.574	4.527
	*COL1A2*	6.364	6.374	6.247	6.409	6.006	6.515	6.305
	*DCN*	9.446	8.636	14.352	7.316	21.372	4.217	10.651
	*ELN*	6.703	6.704	6.702	6.704	6.705	6.706	6.704
	*RUNX2*	5.670	5.267	5.243	5.813	4.777	5.988	5.379
	AVERAGE MAD	6.332	5.959	7.147	6.098	8.969	5.600	6.713
